# Comprehensive genomic profiling of neuroendocrine neoplasms of the colorectum

**DOI:** 10.3389/fgene.2026.1792341

**Published:** 2026-05-12

**Authors:** Hongfa Xu, Xiaoqin Jin, Man Chen, Shaoxiu Yang, Guanghong He, Lirong Ou, Tuhua Li, Qilian Liang, De Cai

**Affiliations:** 1 Department of Oncology, Affiliated Hospital of Guangdong Medical University, Zhanjiang, China; 2 Department of Clinical Pharmacy, Affiliated Hospital of Guangdong Medical University, Zhanjiang, China

**Keywords:** colorectal, comprehensive genomic, evolve, neuroendocrine tumors, somatic mutation

## Abstract

Neuroendocrine tumors (NETs) are classified into G1, G2, and G3, corresponding to low-grade, intermediate-grade, and high-grade tumors, higher grades show greater aggressiveness and poorer outcomes, with molecular mechanisms remaining unclear. This study aimed to investigate gene mutations across G1, G2, and G3 in 24 colorectal neuroendocrine tumors (CRNETs) using whole-exome sequencing to identify somatic single nucleotide polymorphisms (SNPs). The results showed a prominent T>C single nucleotide variant (SNV) in G1 samples, while C>T was prevalent in G2 and G3. The analysis of significant mutated genes revealed that *HYDIN* (100%) was present in all grades, though mutation sites and frequencies differed. Particularly, tumor-associated HYDIN mutations were exclusive to G3, suggesting it may serve as a candidate biomarker for distinguishing high-grade from lower grades. Important copy number variations (CNVs) were identified in genes such as *POP4* (G1), PPARG, *MYC*, and *F10* (G2), as well as *VOPP1* and *UGT2B17* (G3). The *KMT2A* gene, associated with potential clinical drug responses, exhibited specific mutation sites across all samples. The study identified seven primary mutation signatures, with signature 6 predominant in G3, and highlighted a link to carcinogenic pathways like RTK-RAS, Notch, WNT, and Hippo, thus providing valuable insights into the pathogenesis of CRNETs.

## 1 Introduction

Neuroendocrine Tumors (NET) are rare neoplasms characterized by neuroendocrine differentiation and expression of neuroendocrine markers. They can arise in virtually any anatomic site, with the gastrointestinal tract and pancreas being the most frequent primary locations (Yao et al., 2008; Dasari et al., 2017). According to the 2010 World Health Organization classification scheme, differentiated neuroendocrine tumors are divided into three different grades: G1, G2, and G3, corresponding to low-grade, intermediate-grade, and high-grade. Studies indicate that the prognosis of Pancreatic Neuroendocrine Tumors (PanNETs) correlates with tumor grade, where G2 PanNETs are more aggressive than G1 tumors, and G3 PanNETs exhibit the highest aggressiveness (Rindi et al., 2018). Histologic grades are dependent on mitotic counts and the Ki-67 labeling index. To date, pathologic studies use immunohistochemical (IHC) analysis of biopsy tissue, including the number of nuclear divisions per 2 mm^2^, the Ki-67 index, chromogranin A (CgA), synaptophysin, and neuron-specific enolase, as the first-line method to diagnose the disease. Additionally, auxiliary tools include ultrasound, computed tomography (CT), magnetic resonance imaging (MRI), endoscopy, and serum tumor markers. Particularly, the diagnosis of G3 NETs mainly relies on the characteristics of pathology, including good differentiation, small cellular atypia, and less local necrosis. G1/G2 components may also be detected in background components, suggesting that G3 NET may have evolved from G1/G2 (Liu et al., 2021). Despite these diagnostic tools, the histogenetic classification and molecular landscape of NETs remain poorly defined, significantly hindering the development of targeted therapies. Thus, deciphering the molecular signatures driving NET progression is critical to advancing precision medicine.

Somatic mutations in pancreatic NETs have been well characterized. In contrast, little is known about the molecular drivers of neuroendocrine carcinomas in the gastrointestinal system, for which few specimens have been available for analysis. One analysis revealed that G3 NETs had gene expression profiles that did not easily segregate by organ, shared mutations in *TP53, RB1, APC, CDKN2A*, and the *CDK4*/six-cell cycling pathway, and harbored two somatic copy number alterations that could have prognostic potential across tissue types (Scarpa et al., 2017). Poorly differentiated colorectal neuroendocrine carcinomas frequently harbor *BRAF* mutations, which are identified in both pure poorly differentiated neuroendocrine carcinomas and poorly differentiated neuroendocrine carcinomas with a signet ring cell adenocarcinoma component. One study compared neuroendocrine differentiation and *KRAS/NRAS/BRAF/PIK3CA/TP53* mutational status in primary and metastatic Colorectal cancer(CRC) and found that neuroendocrine-negative CRC showed a high rate of *NRAS, BRAF, TP53* (comprising primary and metastatic tumor tissue), and *PIK3CA* mutations(only comparing lymph node metastases). Neuroendocrine differentiation of tumor tissues is an important prerequisite for new targeted therapies (Sun et al., 2022). In addition, abnormally regulated Notch, Wnt, and *RAS/RAF/MEK/ERK* signaling are the main pathways in rectal NETs, although aberrant mTOR signaling is also involved (Song et al., 2016). Currently, standardized therapeutic consensus for colorectal neuroendocrine tumors (CRNETs) remains suboptimal. Systemic chemotherapy for advanced cases largely adopts the platinum-based backbone used in small cell lung cancer (SCLC), specifically the etoposide plus platinum (EP) or irinotecan plus platinum (IP) regimens (Shah et al., 2018). While the EP regimen (Cisplatin/Carboplatin + Etoposide) is the global first-line standard, the IP regimen (Cisplatin + Irinotecan) is frequently employed as a potent alternative, particularly in East Asian clinical settings or as a second-line rescue therapy.

We performed whole-exome sequencing (WES) on 24 treatment-naïve CRNETs (G1 = 6, G2 = 6, G3 = 12) to characterize their mutational landscape. Our analysis integrated somatic SNVs, significantly mutated genes (SMGs), and copy number variations (CNVs) across histological grades Our findings characterize the mutational landscape of CRNETs and identify genomic alterations that may be relevant to disease diagnosis and prognosis.

## 2 Materials and methods

### 2.1 Patient tissue collection, processing

FFPE tumor samples from 24 patients with CRNETs were collected from the Affiliated Hospital of Guangdong Medical University, and all cases were pathologically confirmed. Clinicopathological data, including age, sex, tumor location, TNM stage, Ki-67 index, synaptophysin (Syn), chromogranin A (CgA), and survival information, were obtained from medical records. Progression-free survival (PFS) was used as the study endpoint. Patients were included if they had pathologically confirmed colorectal neuroendocrine tumors and were followed up until death or the end of the study. Tumor staging was based on the 8th edition of the American Joint Committee on Cancer (AJCC) system. This study was approved by the institutional ethics committee, and written informed consent was obtained from all patients.

### 2.2 Immunohistochemistry

Samples were stained using standard IHC procedures. Typical neuroendocrine markers, such as synaptophysin, chromogranin A, CD56, CD57, and neuron-specific enolase were used to confirm neuroendocrine differentiation, particularly in case of a doubt based on unusual or variant morphologic features. Specimens were stratified into World Health Organization (WHO) grade groups (G1/G2/G3) according to differentiation criteria. We used P53, RB1, KRAS, and other antibodies and performed hematoxylin and eosin (HE) staining. A semi-quantitative method was used for IHC scoring, and staining intensity was categorized into four grades: 0 (no staining), 1 (weak staining), 2 (moderate staining), and 3 (strong staining). The percentage of positive cells was recorded. The proportion of positive cells was scored from 1 to 4 based on predefined percentage ranges. The immunohistochemistry score was calculated by multiplying the positive area value by the intensity score. A total score >0 was defined as positive expression. The mitotic count and Ki-67 index were used to determine WHO grade: G1 (<2 mitoses/10 HPFs and Ki-67 < 3%), G2 (2–20 mitoses/10 HPFs or Ki-67 3%–20%), and G3 (>20 mitoses/10 HPFs or Ki-67 > 20%). In cases of discordance between the mitotic grade and Ki-67 index, a higher value was used to assign the final grade. The Ki-67 proliferative index is generally of a higher grade in cases in which there is discordance with the mitotic count (Patel et al., 2019).

### 2.3 DNA libraries preparation

Subsequently, genomic DNA was extracted from tumor specimens for whole-exome sequencing (WES) library preparation. The purity of the DNA was assessed using NanoDrop assays, and the DNA concentration was determined using Qubit 3.0. Agarose gel electrophoresis was used to evaluate the quality of the DNA bands, and an Agilent 2100 Bioanalyzer (Agilent, Santa Clara, United States of America) was used to confirm the length and integrity of the DNA. In summary, 1 μg of genomic DNA was fragmented by shearing, then purified, end-repaired, phosphorylated on the five ends, adenylated on the three ends, ligated using indexed pair-end adaptors, purified once more, and amplified via PCR. Agilent SureSelect Target Enrichment Kit Human All Exon V6 Probes (Agilent; 5190-8864) were used for exome capture. The processes of PCR amplification, purification, library validation, normalization, and pooling were executed using the xGen Hybridization and Wash Kit (IDT, 1080584). Library concentrations were determined using the Equalbit® dsDNA HS Assay Kit, followed by library fragment distribution analysis using the Agilent 4200 TapeStation System and precise determination of library molarity using the KAPA Library Quantification Kit (Illumina) universal qPCR mix. The libraries underwent sequencing on the NovaSeq 6000 platform using the NovaSeq S4 reagent kit, resulting in the generation of 300 base pair reads (2 × 150) from the terminal library fragments.

### 2.4 WES data analysis

The procedures previously outlined were followed for adapter trimming, Burrows-Wheeler Aligner (BWA) read mapping, and Genome Analysis Toolkit (GATK) processing (Xiao et al., 2021). The Sequence Read Archive database (https://www.ncbi.nlm.nih.gov/sra,(accessed May 2025)) received the generated BAM files. We uploaded the WES data of CRNETs G1/G2/G3 as separate data. VarScan 2 software (Koboldt et al., 2012) was used as a somatic mutation invoker, which can be configured to detect variants at low frequencies, reducing the overall false-positive rate. SNPs and insertions and deletions (indels) were functionally annotated using VEP and SnpEff (version 1.9.6) software (Cingolani et al., 2012; Hunt et al., 2022) utilizing data from publicly accessible databases, such as ClinVar and Exome Aggregation Consortium (ExAc) (http://exac.broadinstitute.org,(accessed May 2025)). Filtering methods were applied to identify variants that were clinically relevant and present in affected individuals at minor allele frequencies (1%) in 1000 samples using the Genomes Project, Single Nucleotide Polymorphism (dbSNP), ExAC, and Exome Variant Server databases.

### 2.5 Summary of WES data analysis

The CIRCOS figure, generated using ClicO FS (Cheong et al., 2015), illustrates the genome-wide distribution of mutations. Maftools (Mayakonda et al., 2018) facilitates the analysis of six categories: SNVs, SNV proportions, oncoplots displaying mutational frequency, lollipop plots for highly mutated genes, and mappings of mutually exclusive or co-occurring gene sets. Domain enrichment analysis is conducted using Pfam. TBtools allows for the efficient creation of various visualizations, including Venn diagrams, heatmaps, and Circo’s graphs (Chen et al., 2020).

### 2.6 Pathway enrichment analysis

We downloaded pathway data from ConsensusPathDB (http://cpdb.molgen.mpg.de,(accessed May 2025)). An open-source online collection of pathways that incorporate 32 sources, including Kyoto Encyclopedia of Genes and Genomes (KEGG), Wikipathways, Protein Data Bank (PDB), and Reactome. Applications comprise overrepresentation analysis to characterize diverse sets of molecules, gene set enrichment analysis, and identification of upstream regulators, spanning various biological contexts. KEGG pathway enrichment analysis was performed using ConsensusPathDB to identify the main functional pathways of CRNETs (Herwig et al., 2016).

### 2.7 SMG analysis

To identify SMGs in 24 CRC-NET samples, we used the MutSigCV algorithm (Lawrence et al., 2013), which adopts a comprehensive background mutation model (not a fixed rate) to estimate each gene’s expected mutation frequency for accurate SMG identification. It was performed using default covariate expression levels and DNA replication timings. Statistical significance was assessed by comparing the observed mutations in a gene to the expected counts derived from the background model. The mutation effect refers to a broad class of effects that the mutation exerts on the gene. We analyzed the VAF distribution across all samples to assess the reliability of somatic mutation calls. Median VAFs for INDELs were 10.2%, 11.4%, and 10.8% in G1, G2, and G3, respectively, with most variants ranging from 5% to 34∼38%. For SNPs, median VAFs were 12.8%, 15.3%, and 19.0%, respectively, with the majority clustering between 5% to 40%, suggesting that our computational filtering effectively enriched for somatic events.

### 2.8 CNV analysis

We determined the total amount of sequencing reads for every exon in every sample and gene using BAM files that contained the exons’ genomic positions from the UCSC Genome Browser (https://genome.ucsc.edu,(accessed June 2025)). The average sequencing depth of the samples was used to adjust the data. To prevent false positives, we concentrated on genes with more than two exons. Genes were classified as having copy number gain if every exon had a copy number greater than 2.8. In a similar vein, genes were identified as suffering copy number loss if a copy number <1.2 was present in every exon. The Oncoprinter Software was utilized to visualize the distribution of genes exhibiting CNVs across the sample cohort, as previously outlined (Gao et al., 2013). Somatic mutation prediction: Potential somatic SNVs were predicted using ISOWN, a reliable method that distinguishes germline polymorphisms from somatic mutations in cancer cells when corresponding normal tissues are not available. VCF format files validated by the database were annotated using COSMIC (v69), dbSNP (v142), ExAC (v2), PolyPhen WHESS (2015 version), and Mutation Assessor (2013 version) with default criteria. ISOWN was used to predict somatic mutations with low VAF. We then examined somatic mutations in more detail using SomVarIUS—an additional single-sample variant caller—in conjunction with ISOWN (Kalatskaya et al., 2017; Smith et al., 2016).

### 2.9 Mutation signature analysis

Mutalisk (http://mutalisk.org,(accessed June 2025)) facilitated the straightforward analysis of somatic mutation signatures within somatic SNVs, obviating the requirement for intricate methodologies or preparatory protocols. It could identify mutational signatures that we conventionally expect from a specific somatic tissue. A linear regression test was applied as the maximum likelihood estimation method for the decomposition of mutation signatures referring to the 30 COSMIC signatures. To avoid overfitting, candidate models with different numbers of contributing signatures were evaluated using the Bayesian Information Criterion (BIC), and the optimal model with the best balance between goodness-of-fit and model complexity was selected (Lee et al., 2018).

### 2.10 Statistical analysis

SPSS 26.0 was used to create survival analysis illustrations. The Kaplan-Meier curve was plotted to describe the progression-free stage of the patient, and the log-rank test was used to compare the differences in the progression-free stage of patients in different groups. The log-rank test’s p-values and hazard ratios were also computed.

## 3 Results

### 3.1 Immunohistochemistry and clinically relevant information

We collected clinical information and pathological specimens from 24 patients with colorectal neuroendocrine tumors. The patients were divided into G1, G2, and G3 stages based on the degree of tumor differentiation. The detailed information can be found in Supplementary Figure S1 below. The progression-free survival of G1 was higher than that of the other two groups, with G3 showing the worst survival trend, although the difference was not statistically significant, due to the quantities of samples (P = 0.312) (Supplementary Figure S2). The lack of statistical significance in PFS likely reflects the limited power of the current study rather than a lack of biological relevance. The observed trends still warrant further investigation. Based on previous studies, mutations in TP53, KRAS, and RB1 were more common in high-grade gastroenteropancreatic neuroendocrine neoplasms (Puccini et al., 2020; Venizelos et al., 2021). Thus, all samples were stained with P53, RB1, and KRAS antibodies for immunohistochemistry. Diffuse positivity for P53, RB1, and KRAS was seen in all three types of samples, G1, G2, and G3, with a higher prevalence of these positives in G3 compared to G2 and G1, which is consistent with previous findings (Supplementary Figure S3a-d).

### 3.2 Characteristics of mutations and SMGs

**FIGURE 1:**
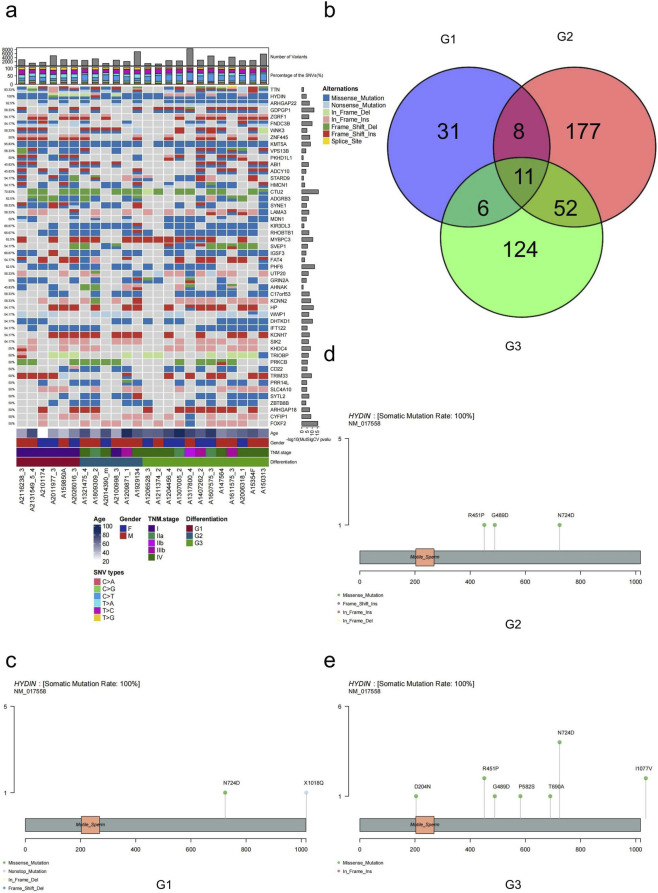
Characteristics of mutations in CRNETs. **(a)** Name, mutation frequency, distribution in CRNETs samples, and p value of significance of the SMGs with a MutSigCV p value < 0.05. Histograms of the total mutation number and fraction of the six types of SNVs in each CRNETs sample are also shown. **(b)** Overlap of SMGs detected in the G1,G2,G3 Colorectal neuroendocrine cancers. **(c–e)** Different mutation sites of HYDIN in G1, G2 and G3. Known hotspot mutation sites in tumors are labeled. The motile sperm labeled box indicates the annotated functional domain of the HYDIN protein.

We acquired average sequencing depths ranging from 160 to 200× for CRNETs belonging to grades G1, G2, and G3 in order to examine the mutations (Supplementary Figure S4a). Furthermore, we used the Circle chart to illustrate the genome-wide distributions of each sample, including chromosome number and location, detailed somatic mutations, and indels (Supplementary Figure S4b). We observed that as the degree of differentiation decreased, the number of SNVs and insertions/deletions (INDEL) mutations increased. There was a more pronounced increase in the number of SNVs in G3, and the number of somatic mutations exceeded the number of INDEL mutations (Supplementary Figure S5a-d). Among the six mutation types (C>T, C>A, C>G, T>A, T>C, T>G), the C>T mutation accounted for the highest proportion of base conversions among the six types in all samples. Using ISOWN and SomVarIUS software to identify somatic point mutations. The C>T mutation predominated among the six types of SNVs, representing 27.79% of the total (Supplementary Figure S6a). An analysis of the distribution of observed and factorized signatures of all SNVs across the 96 potential mutation types revealed a high cosine similarity of 0.931 and a BIC of 72894.850, verifying the dominant somatic SNV of C>T (Supplementary Figure S6b). T>C was the top SNV for G1 samples, while C>T was the top SNV for G3 samples (Figure 1a; Supplementary Figure S7a-c). Furthermore, We analyzed somatic mutations across the genome in G1, G2, and G3 samples to identify regions with hypermutations. In the G1 and G2 samples, the hypermutated region may have been located on chromosome 17 (Supplementary Figure S6c,d). In the G3 samples, the hypermutated region may be located on chromosome 19 (Supplementary Figure S6E). In the single nucleotide variant, further analysis revealed that transitions were more prevalent in G1 samples, while transversions were more predominant in G3 samples (Supplementary Figure S6d-f). At the same time, we detected nine types of mutations, including frameshift deletion, frameshift insertion, nonframeshift deletion, nonframeshift insertion, synonymous SNV, nonsynonymous SNV, stopgain, stoploss, and splicing, where nonsynonymous SNV has an absolute advantage. In the G3 sample, reduced differentiation was associated with a substantial increase in mutation counts, with nonsynonymous and synonymous SNVs ranking as the top two categories (Supplementary Figure S8a-d). Notably, when we detected SMGs in CRNETs, we found that HYDIN showed the highest mutation frequency of 100% (24/24) in all patients, and the main variant types were missense mutations. Moreover, we referenced our findings with the cBioPortal (TCGA, Colorectal Adenocarcinoma cohort, COSMIC, Large Intestine - Carcinoma cohort, ICGC, Colorectal Cancercohort) and found that the HYDIN mutation frequency is approximately 10%–15% (Supplementary Table S1a). However, we emphasize that public datasets like TCGA predominantly consist of colorectal adenocarcinomas, whereas our cohort is exclusively comprised of colorectal neuroendocrine tumors. The distinct histological and molecular landscape of neuroendocrine neoplasms compared to common adenocarcinomas likely accounts for this significant discrepancy.

Therefore, this gene may serve as a specific candidate marker for neuroendocrine tumors, and its specific mechanism needs further study. *KMT5A* had the second-highest mutation frequency of 95.83% (23/24); all variant types were missense mutations. The nomenclature, mutation frequencies, sample distributions, and statistical significance associated with these SMGs are succinctly presented (Figure 1a). MutSigCV identified 409 candidate SMGs in CRNETs. SMGs differed in the three levels of differentiated CRNETs and shared 11 significant levels of common genes; among them, the *HYDIN* mutation occupied the greatest predominance (Figure 1b). Furthermore, we identified the distinct mutation loci of *HYDIN* in G1, G2, and G3. They were displayed in the various functional domains as shown in Figures 1c–e (Figures 1c–e). This further suggested as a candidate recurrently mutated gene, HYDIN may involvement in CRNET malignancies.

### 3.3 Pathway analysis associated with mutant genes

We further investigated the enriched signaling pathways associated with the mutated genes. The commonly mutated genes in the G1 and G2 samples were mostly enriched in the ECM-receptor interaction signaling pathways, while those commonly mutated in the G2 and G3 samples were mainly enriched in the Focal adhesion pathways. The common genes among G1, G2, and G3 samples were predominantly enriched in the MAPK signaling pathway (Supplementary Figure S9a-c).

Moreover, we further detected the SMGs’ relative signaling pathway. In the G1 and G2 samples, the SMGs were specifically enriched in cancer choline metabolism, while those in the G2 and G3 samples were enriched in the mTOR signaling pathway (Supplementary Figure S9d,e). These results suggest that the PI3K-Akt, MAPK signaling pathways, mTOR signaling pathways, and other pathways may contribute to neuroendocrine tumor development, the specific causal mechanisms remain to be elucidated through functional validation. The information showed that there were similarities and differences in the signaling pathway-related gene alterations between the G1, G2, and G3 populations. This finding suggests unique characteristics of the mutated pathways at different degrees of differentiation.

### 3.4 Distinct mutational statuses of known cancer-related genes

**FIGURE 2:**
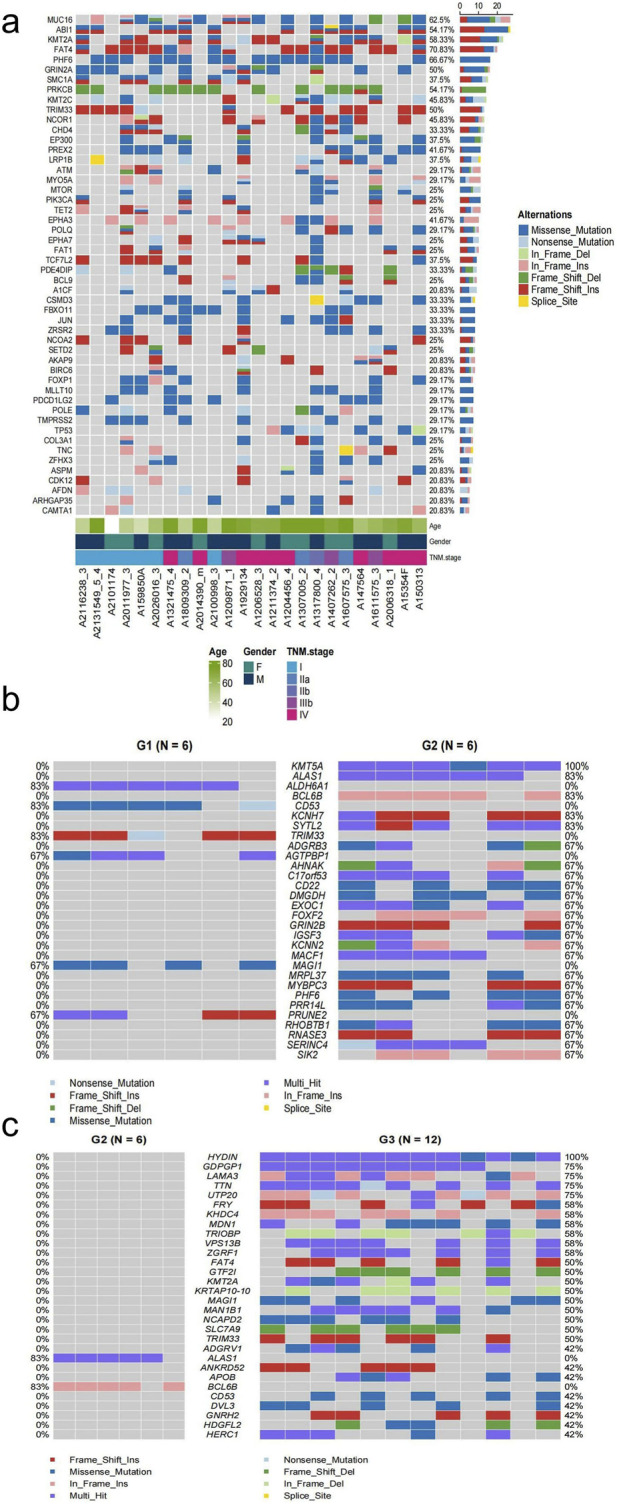
Distinct mutational statuses of known cancer-related genes. **(a)** Mutations in known cancer-related genes in the CGC database that were detected in more than five CRNETs sample. **(b)** Mutations in genes mutated specifically in G1 or G2. **(c)** Mutations in genes mutated specifically in G2 or G3.

To elucidate the oncogenic mutations in NETs, we analyzed mutational profiles using the COSMIC Cancer Gene Census (v19) database. In our cohort of 24 CRNETs, we identified recurrent alterations (≥5 cases) in 50 cancer-associated genes (Figure 2a). The most frequently mutated gene was *FAT4* (70.83%), with 20 other genes showing a frequency of mutations ≥30%, including *MUC16* (62.5%), *KMT2A* (58.33%), *PHF6* (66.67%), and others. We found that the frequency of *FAT4* mutations was relatively high in G1, G2, and G3 samples separately, suggesting that *FAT4* may be a candidate driver gene for colorectal neuroendocrine tumors. Furthermover, we evaluated FAT4 expression in normal tissues using the GTEx. FAT4 was found to be expressed across multiple normal tissues (Supplementary Table S2), particularly in gastrointestinal and lung tissues. For another, we also compare samples from G1, G2, and G3 to detect mutations in genes associated with tumors. In contrast, 24 gene mutations, including *KMT5A* (100%), were found in G2 but not in G1 (Figure 2b). In addition, 28 genes, including *HYDIN* (100%), exhibited tumor-associated mutations in G3, while none were found in G2 (Figure 2c). We describe the transition from the G1 to the G2 phase may be significantly influenced by *KMT5A*. Tumor-associated mutations in the *HYDIN* gene may be crucial for the transition from the G2 phase to the G3 phase.

### 3.5 CNVs

**FIGURE 3:**
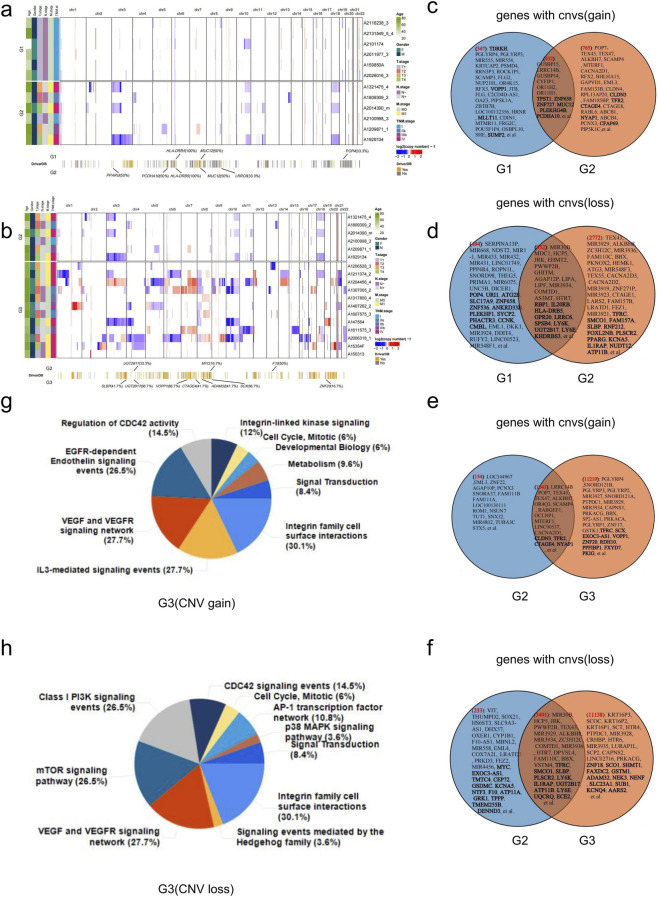
Genes with CNVs detected in CRNETs. **(a)** Genome-wide distribution of all the CNVs in each sample of the G1 and G2 samples accompanied by the clinical information of the samples. **(b)** Genome-wide distribution of all the CNVs in each sample of the G2 and G3 samples In addition, the genes with high-frequency potential driver CNVs in G1 and G2 samples were labeled. **(c,d)** Overlap of genes with a copy number gain **(c)** and loss **(d)** detected in G1 and G2 samples. The name of genes with high-frequency CNVs was labeled in the Venn figures. Genes with potential driver CNVs were also labeled and highlighted in bold font. **(e,f)** Overlap of genes with a copy number gain **(e)** and loss **(f)** detected in G2and G3 samples. **(g)** Results of pathway enrichment analysis on the genes with a copy number gain in G3. **(h)** Results of pathway enrichment analysis on the genes with a copy number loss in G3.

Comparative analysis of CNVs across tumor grades (G1-G3) demonstrated significant heterogeneity in both the frequency and genomic distribution of these alterations. Specifically, G1 *versus* G2 comparisons identified recurrent CNVs in several candidate driver genes, including *HLA-DRB5*, *PPARG*, *MUC12*, *POP4*, *PCDHA*, and *LRRC6* (Figure 3a). In G2 and G3 samples, the high-frequency CNVs were *F10*, *UGT2B17*, *MYC*, *SCX*, *VOPP1*, *ZNF20*, *CTAGE4*, *ADAM32*, and *SLBP* (Figure 3b). In our cohort, A total of 34,037 genes with copy number variations were identified in this study, including 2,225 in the G1 group,4,921 in the G2 group, and 26,891 in the G3 group (Supplementary Table S3). Subsequently, we scrutinized the genes exhibiting CNVs in G1 and juxtaposed them with those in G2. Out of the 1197 genes identified with copy number gains in G2, 432 genes overlapped with those in G1. Similarly, among the 3724 genes displaying copy number losses in G2, 952 genes were common with those in G1. Similarly, when comparing G2 to G3, we observed that G3 had 12262 genes with an increased copy number. Of these, 1043 genes were identified in the G2 group. Additionally, G3 had 14629 genes with copy number loss, with 3491 genes shared with G2. The high-frequency gene CNVs and potential driver CNVs were highlighted in bold font (Figures 3c–f). Functional enrichment analysis of genes with copy number gain in G3 samples revealed several cancer-related pathways, such as integrin family cell surface interactions, VEGF and VEGFR signaling networks, and IL3-mediated signaling events (Figure 3g). The enrichment signaling pathway for copy-number deletion genes in group G3 is illustrated in Figure 3h. Several cancer-related signaling pathways have been identified, including the mTOR signaling pathway, class I PI3K signaling events, integrin family cell surface interactions, and the VEGF and VEGFR signaling networks. Our results demonstrate the potential for high-frequency copy number mutations in the NET. Additionally, as differentiation decreases, copy number variant genes become more enriched in tumor-related signaling pathways.

### 3.6 Clinical relevance of genes and pathways with genomic alterations

**FIGURE 4:**
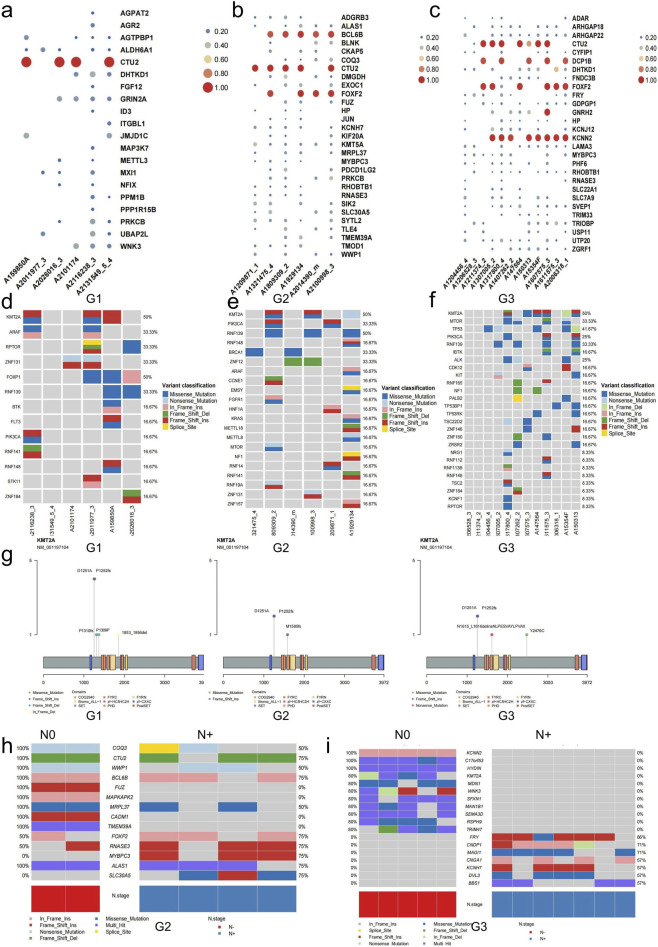
Clinical relevance of mutated genes in CRNETs. **(a,b,c)** Genes with disease-related mutations detected in G1 **(a)**, G2 **(b)**, G3 **(c)**. **(d–f)** Detailed mutation information on genes with potential clinically actionable mutations recorded in the OncoKB database about G1 **(d)**, G2 **(e)**, G3 **(f)**. **(g)** Detailed information on the mutation locations of the most frequently mutated gene in clinically relevant genetic mutations in G1, G2, and G3. **(h,i)** Genes mutated specifically in stage N0 and N+ in G2 **(h)** and G3 **(i)**.

To assess the potential clinical correlation to these mutations, our initial focus was on the underlying functions and established disease associations in the ClinVar database. We tested the disease-related mutations in samples G1, G2, and G3, and found 20, 77, and 93 genes with disease-related variants separately (Supplementary Figures S10a,b), in which G1 and G2 had seven common disease-related variant genes. Similarly, G2 and G3 had 20 common disease-related variant genes. We found that the *CTU2* gene had a high mutation frequency in all three samples; the *FOXF2* gene mutation only appeared in the G2 and G3 samples, accounting for the top few mutations, while the high mutation frequency of *DCP1B* and *KCNN2* was just mutated in the G3 samples (Figures 4a–c). These results suggest that *CTU2* may be an essential gene for tumorigenesis of colorectal neuroendocrine tumors, and *FOXF2* may be an important gene that causes the progression of G1 to G2 and G3. For another, NET may gradually proceed to the G3 type due to the involvement of *DCP1B* and *KCNN2* as critical genes (Figures 4a–c). Subsequently, we examined the clinical correlation to the enriched signaling pathways that exhibited specific mutations in G2 samples compared with those in G1 samples (Supplementary Figure S10c), including the cAMP, VEGF, axon guidance, and osteoclast differentiation pathways. Additionally, axon guidance, VEGF, ErbB, mTOR, hepatocellular carcinoma, choline metabolism in cancer, and focal adhesion pathways were more enriched in G3 than in G2 (Supplementary Figure S10d). Our findings suggest that with the increase of tumor grade, tumor-associated mutant genes are more enriched with signaling pathways such as the ErbB signal pathway, mTOR signal pathway, etc.

In the OncoKB dataset, a total of 57 actionable genes were identified, among which 13, 21, and 26 displayed potential drug responses in samples classified as G1, G2, and G3, respectively. Notably, *KMT2A* and *RNF139* exhibited consistently high mutation frequencies across the three distinct pathological subtypes of NETs; in addition, TP53 shows high mutation frequency only in G3. We found both identical and different mutation sites in the above identical mutated genes (*KMT2A* and *RNF139*) in the three different degrees of differentiation samples (Figures 4d–g; Supplementary Figure S11a). The mutations *TP53* A159D, R175H, R123X, and 127_128del are displayed in Supplementary Figure S11b. There is promising clinical data in patients with *KMT2A* fusion-positive acute myeloid leukemia treated with the menin inhibitor SNDX-5613, but there have been no relevant studies in neuroendocrine tumors. TRC8 (*RNF139*) E3 ligase can recognize and effectively degrade CD112. Inhibition of CD112 ubiquitination can increase its expression on the surface of tumor cells, thereby enhancing the sensitivity of tumor cells to NK cytotoxicity and boosting the killing effect of NK cells on tumor cells. The above genes provide directions for the study of target drugs related to the treatment of neuroendocrine tumors.

We also examined the variations in altered genes throughout distinct N stages in tumors with different levels of differentiation in CRNET. In the G2 samples, mutations were found in *FUZ, MAPKAPK2, CADM1*, and *TMEM39A* only in the N0 group (n = 2). Additionally, mutations in *MYBPC3* and *SLC30A5* were only observed in the N1-2 group (n = 4) (Figure 4h). In G3 samples, *KCNN2, C17orf53, HYDIN, KMT2A, MDN1, WNK3, SFXN1, MAN1B1, SEMA3D, RSPH9*, and *TRIM47* were only mutated in the N0 group (n = 6). In contrast, *FRY, CNDP1, MAGI1, CNGA1, KCNH7, DVL3,* and *BBS1* were mutated within the N1/N2 group (n = 7) (Figure 4i). The above-mentioned genes with relevant mutations at the N+ level that may cause tumor progression and metastasis still need to be further investigated.

### 3.7 Mutational signatures and oncogenic signaling

**FIGURE 5:**
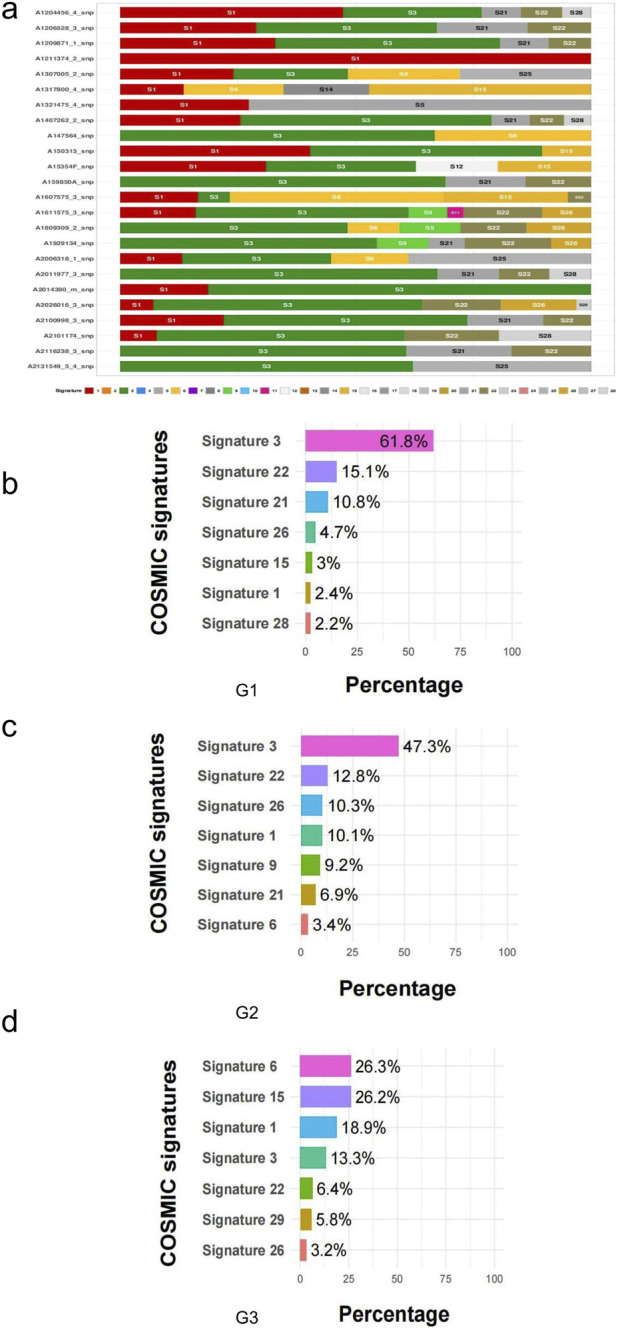
Characteristics of potential somatic SNVs. **(a)** The mutation signatures discovered in each CRNET samples. **(b–d)** Known COSMIC mutation signatures discovered in the potential somatic SNVs in G1 **(b)**,G2 **(c)**,G3 **(d)**.

We detected mutation signatures in potential somatic SNVs. The decomposition analysis used 30 cosmic features and combined the BIC for the ranking analysis. The mutation signatures were diverse among the 24 CRNET samples (Figure 5a). For the decomposed signatures of potential SNVs in the NET, Signatures 3 and 22 contributed to the largest component in G1 and G2 samples, while Signatures 6 and 15 contributed to the largest component in G3 samples (Figures 5b–d).

**FIGURE 6:**
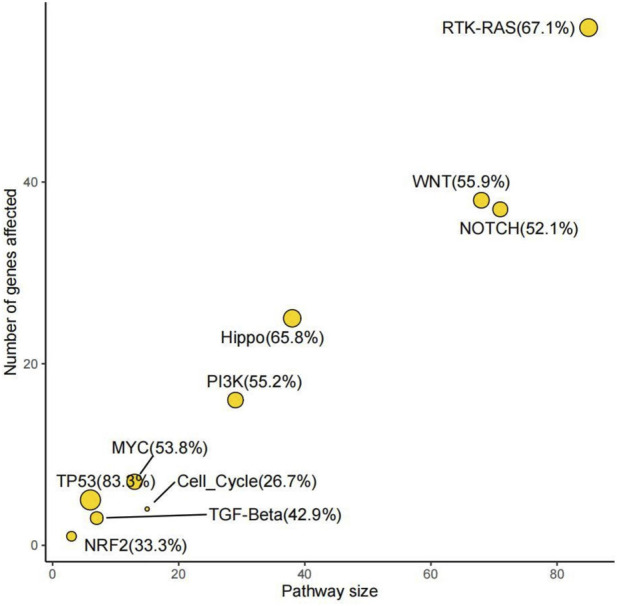
Enrichment analysis of known oncogenic signaling pathways in TCGA cohorts. The horizontal axis represents the total number of genes in the pathway and the vertical axis represents the number of genes mutated in the corresponding pathway. The size of the circle represents the percentage of mutated genes in all genes of the pathway, the detailed number of which is also labeled after the pathway name.

Lastly, the signature of somatic SNVs and CNVs associated with the pivotal members of ten critical oncogenic pathways in TCGA cohorts was investigated. Our study revealed the significant signal pathway profile of mutated genes originating from CRNET, demonstrating a high enrichment of CRNET mutated genes in tumor-related pathways. Specifically, more than half of the genes were found in seven pathways. Furthermore, it was observed that the Notch, WNT, Hippo, and RTK-RAS pathways each harbored a considerable number of mutated genes, exceeding 20 in count. Collectively, these data suggest that the primary form of somatic mutation occurs in several oncogenic pathways, including the Notch, WNT, Hippo, and RTK-RAS (Figure 6).

## 4 Discussion

We successfully recruited 24 cases of CRNETs from the clinical practice site, where samples were divided into G1, G2, and G3 according to different degrees of differences, examined somatic mutations and copy number variations in all the samples, identified potential somatic SNVs, and conducted an analysis of somatic mutational signaling and oncogenic signaling pathways in CRNETs. These results contribute to our comprehension and offer worldwide perspectives on the genetic profiling of CRNETs, which may inform clinical assessment and prognosis approaches.

Analysis of SMGs revealed ubiquitous HYDIN alterations (100% prevalence across all tumor grades), establishing its potential as a diagnostic biomarker for neuroendocrine neoplasms. Notably, *HYDIN* demonstrates the highest mutational frequency among known cancer-associated genes in melanoma cohorts. Furthermore, gene set enrichment analysis revealed significant enhancement in DNA repair and antitumor immunity in patients with *HYDIN* mutations (Li et al., 2023). In the comparison of tumor-associated mutant genes between G2 and G3 samples, it is noteworthy that the tumor-associated mutations in the *HYDIN* gene were identified exclusively in the G3 samples.

Furthermore, mapping the mutation sites of the *HYDIN* gene across G1, G2, and G3 samples revealed a shared mutation site (N724D). Additionally, G3 samples exhibited more differential mutation sites (such as D204N, I1077V, P582S, and T690A) than G1 and G2 samples. *HYDIN* is a protein-coding gene. Diseases associated with *HYDIN* include ciliary dyskinesia. Primary, 5, and Primary Ciliary Dyskinesia. Primary cilia (PC) are frequently observed in small cell lung cancers (SCLCs), but not in non-small cell lung cancers (non-SCLCs) or lung carcinoids. The pathobiological characteristics of their PCS display a range of specific features. This indicates a significant connection between the development of lung cancer and PC. Previous investigators identified *HYDIN* mutations in samples from patients with primary ciliary dyskinesia (PCD) and discovered homozygous c.3985G>T mutations affecting an evolutionarily conserved splicing receptor site. Additionally, whole exome sequencing revealed a homozygous HYDIN mutation, c.922A>T (p.Lys307 (*) (Olbrich et al., 2012; Shinmura et al., 2023). Studies have found that patients with primary ciliary dyskinesia (PCD) exhibit new complex heterozygous splicing variants of HYDIN (c.5969–2A>G, c.6316 + 1G>A) as well as deleterious complex heterozygous missense variants (c.9507C>G and c.14081G>A). These alterations affect the common splicing receptor sites of HYDIN, leading to progressive sperm motility issues, morphological abnormalities, and inferior lobe bronchiectasis (Yu et al., 2023). These findings suggest that the *HYDIN* gene may serve as a potential biomarker for diagnosing CRNETs, and its mutations could be a driving force behind the malignant progression of the disease.

The mutation frequency of the tumor-associated gene *FAT4* was high in all the G1, G2, and G3 samples, indicating that *FAT4* may be as a candidate driver gene for colorectal neuroendocrine tumors. *FAT4* exhibits recurrent mutations in a diverse array of human malignancies, notably including hepatocellular carcinoma (HCC). Additionally, expression profiling has indicated that *FAT4* functions as a tumor suppressor gene in the context of HCC and other cancer types (Katoh, 2012; Mao et al., 2022). Tumor-associated genes were comparatively analyzed among the G1 and G2 samples, revealing 24 gene mutations unique to the G2 samples. As the highest mutation frequency gene among them, *KMT5A* is the only lysine methyltransferase that specifically monomethylates histone H4K20. Aberrant *KMT5A* expression is closely linked to the proliferation, invasion, metastasis, and prognosis of various cancers. Consequently, targeting *KMT5A* warrants investigation as a potential therapeutic vulnerability (Yang et al., 2021). Meanwhile, 28 genes, such as *HYDIN* (100%), whose tumor-associated mutations were altered in G3 but not in G2. Therefore, high mutation levels of mutated *HYDIN* may be potential specific biomarkers for G3. *HYDIN* is a prevalent somatic mutation in colorectal, breast, and other cancers that elicit frequent and coordinated adaptive immune responses; nevertheless, its relevance to ICI treatment has not been studied (Yang et al., 2024; Yim et al., 2021).

When examining the genes about potential drug responses, it appears that G1, G2, and G3 may have mutated genes with similar potential clinical drug responses, such as RNF139, which mutated C427S. Surprisingly, for the gene *KMT2A,* we found both identical and specific mutation sites in three types of samples, such as *KMT2A* D1251A and P1252fs mutations were the same in all samples, but M1585fs mutations were only in G2, Y2476C, and N1615-L1616delinsNLPESVAYLPVAX only in G3. Inhibiting the interaction of histone and non-histone proteins in the *KMT2A* core complex is a promising pharmacological strategy for treating leukemia. *KMT2A* inhibitors, such as tazemetostat, gained approval for epithelioid sarcoma and follicular lymphoma (Castiglioni et al., 2022). Precisely predicting the oncogenic functions and responses of various *KMT2A* mutations to different *KMT2A* inhibitors may have crucial therapeutic implications. Random finger protein 139 (*RNF139*), a membrane-bound E3 ubiquitin ligase, has been confirmed as a tumor suppressor in gliomas; there are relevant target drugs in development (Chen X. et al., 2021).

Several genes with high frequency and important CNVs have been observed in G1, G2, and G3 samples, such as *POP4, MUC12*, and *HLA-DRB5* in G1; *PPARG*, *MYC*, *F10*, *HLA-DRB5*, *MUC12*, *UGT2B17*, *PCDHA10*, and *LRRC6* in G2; and *VOPP1*, *UGT2B17*, *SLBP*, *CTAGE4*, *ADAM32*, *and SCX* in G3; Moreover, we still identified several potential SNVs within the ambit of overlapping CNVs. such as POP4, MUC12, MYC, UGT2B17, LRRC6, CTAGE4 and ADAM32,et ac. This co-occurrence suggests a more profound inactivation of these candidate genes, potentially driving the increased aggressiveness observed in higher-grade CRNETs. These CNVs may serve as potential driver genes. POP4 was significantly overexpressed in primary breast cancer and breast cancer cell lines. Silencing *POP4* selectively reduced the viability of cancer cells undergoing expansion (Natrajan et al., 2012). *MUC12*, *PPARG*, and *MYC* have been implicated in regulating cancer cell viability, proliferation, and differentiation, suggesting their potential utility as independent prognostic indicators for colorectal cancer survival due to their anti-tumor properties (Ogino et al., 2009; Jiang et al., 2019). *HLA-DRB5* expression is primarily associated with prognosis and immune infiltration, with fewer tumor data. *F10* is associated with tumor promotion and is expressed in breast cancer, liver cancer, gastric adenocarcinoma, and other adenocarcinomas (Zhang and Pang, 2017). One meta-analysis suggested that *UGT2B17* polymorphisms are linked to susceptibility to prostate cancer and may increase the risk of developing the disease (Kpoghomou et al., 2013). Queries of the public database of gene expression profiling data (Oncomine) have revealed that *VOPP1* transcripts are highly expressed in several prevalent human cancers, including breast, pancreatic, and lymphoma. The functions of *PCDHA10*, *LRRC6*, *ZNF728*, *ZNF845*, and *ZNF90* in tumorigenesis remain insufficiently documented and warrant additional investigation in forthcoming research endeavors.

We also detected the differences in mutated genes with distinct N stages. No lymph node metastases were seen in the G1 patient due to the high degree of differentiation. In the G2 samples, *FUZ*, *MAPKAPK2* mutations were found only in the N0 group (N = 2). In hepatocellular carcinoma (HCC) and gastric adenocarcinoma, elevated *FUZ* expression is associated with a reduced probability of survival, indicating that *FUZ* may exert a protective influence against the development of tumors. Our findings suggest a correlation between FUZ mutations and the G2 stage of early CRNETs (Chen Z. S. et al., 2021). *MAPKAPK2* is implicated in the growth and invasion of tumors, including colon, bladder, skin, and prostate cancers. Its inhibitors have the potential to serve as anticancer drugs that impede the early stages of cancer development (Soni et al., 2019). In G3 samples, *CNDP1*, *MAGI1*, and *DVL3* were exclusively mutated in the N1/N2 group. *CNDP1* is involved in carnosine metabolism, and decreased plasma levels are associated with a poor prognosis for gastrointestinal cancers (Dai et al., 2023). Clinicopathological and experimental evidence suggests that *MAGI1* and *DVL3* may act as a tumor suppressor, expressed in various cancers, including hepatocellular, colorectal, breast, and gastric cancers (Wörthmüller and Rüegg, 2021; Li et al., 2022). The aforementioned gene mutations require further investigation in CRNETs in the future. These genes may act as hub genes, driving the migration of neuroendocrine tumors.

We predicted seven signatures for somatic mutations in individual G1, G2, and G3 samples. Signature 3 was associated with DNA double-strand break repair failure due to homologous recombination, contributing to the largest component in G1 and G2 samples, while feature 6 was associated with defective DNA mismatch repair and was observed in microsatellite unstable tumors, which contributed to the largest component in G3 samples. This phenomenon is most commonly observed in colorectal and uterine cancers and is associated with a high frequency of small (less than 3 bp) indels in single or polynucleotide repeats. These signature-associated biological processes may contribute to CRNET’s malignant progression.

## 5 Conclusion

In conclusion, we have identified potential somatic SNVs and CNVs and analyzed the somatic mutation signatures and oncogenic signaling pathways in CRNETs. We found that the mutation status of tumor-related genes varies in the CRNET at different differentiation stages. In our study, mutations in the *HYDIN* gene were detected in all samples. Interestingly, the mutation sites and numbers of the *HYDIN* gene significantly differ in the G1, G2, and G3 samples. Notably, upon analysis of tumor-associated mutant genes, tumor-associated mutations in this gene were exclusively observed in G3 samples, suggesting that this gene may serve as a key biomarker to distinguish between high-grade and low- and medium-grade neuroendocrine tumors. High-frequency significant copy number variations were examined in samples from G1, G2, and G3, including *PPARG*, *POP4,* and *MYC*. The analysis revealed distinct mutational patterns of these genes in different stages of CRNETs, suggesting a potential role for these genes as driver genes, their definitive role as drivers still requires future experimental validation in larger, independent cohorts with matched normal samples. Moreover, we examined mutations in the drug-responsive gene *KMT2A* and identified identical and specific mutation sites in three types of samples. This finding indicates that *KMT2A* could. offer valuable insights for research on targeted therapies for CRNET. Finally, our analysis of somatic SNV-carrying genes revealed their enrichment in oncogenic signaling pathways, specifically in RTK-RAS, Notch, WNT, and Hippo pathways. Overall, our study provides important insights into understanding the genetic characteristics, clinical assessment, and treatment of CRNETs, offering valuable references for future research. These findings have significant implications for disease staging and treatment.

In this study, exon sequencing was performed on collected FFPE tumor samples. However, the absence of matched normal tissue FFPE samples from the same patients may have impeded the accurate identification of true somatic variants. For high-frequency mutation analysis, MutSigCV software was utilized, with the 1000 Genomes Project (2015aug_all), Exome Sequencing Project (ESP6500SI-V2), and Exome Aggregation Consortium (ExAC) datasets serving as controls to calculate significant somatic mutations. Additionally, Mutalisk was employed to directly analyze mutational signatures from somatic single-nucleotide variants (SNVs). To further elucidate the associations and distinctions in somatic mutations across different pathological grades of neuroendocrine tumors (NETs), subgroup analyses were conducted: G1 samples served as the control group when comparing G1 and G2, while G2 samples were the reference for G2 vs. G3 comparisons, thereby delineating mutation profiles across pathological grades. Notably, Despite the novel insights into the genomic landscape of CRNETs in our study, several limitations must be acknowledged: Sample Size and Rarity: Sample The cohort size (n = 24) is relatively small, reflecting the clinical rarity of colorectal neuroendocrine tumors. This constraint limited the statistical power for subgroup-specific comparisons (G1 vs. G2 vs. G3) and survival analyses (p = 0.312). Furthermore, ingle-Omic Perspective: Our study relied exclusively on whole-exome sequencing (WES). While WES effectively identifies protein-altering mutations, it does not capture transcriptomic changes, epigenetic modifications, or non-coding regulatory variations that also drive tumor progression. in addition, Validation Gap: the identified candidate drivers, lack direct functional validation in in vitro or *in vivo* models. Our findings are exploratory and hypothesis-generating, potential false-positive findings in these analyses warrant further validation. Given the scarcity of epidemiological data on colorectal NETs in China—where most studies are limited to small single-institution cohorts—our study retains significance as it examines rare tumor samples with deep sequencing-derived mutational data. Future work will expand the sample size and crucially, isolate primary cells from fresh CRNET tissues to validate identified mutations through *in vitro* functional assays and *in vivo* animal experiments to clarify their impact on tumor cell behavior and molecular mechanisms.

## Data Availability

The datasets presented in this article are not readily available because of ethical and privacy restrictions. Requests to access the raw sequence data should be directed to the corresponding authors, subject to a reasonable research request and the signing of a formal Data Transfer Agreement (DTA).

## Supplementary material

SUPPLEMENTARY FIGURE S1 Clinical information of the Colorectal neuroendocrine tumors patients.

SUPPLEMENTARY FIGURE S2 Survival analysis of patients about G1,G2,G3 samples.

SUPPLEMENTARY FIGURE S3
**(a-c)** The IHC results for one sample show staining for HE, P53, RB1, and KRASin G1,G2 and G3.Scale bars in the figures are 100x, and the magnification is 20x. **(d)** Histogram of the proportion of positive genes in CRNET samples about P53,RB1,KRAS.

SUPPLEMENTARY FIGURE S4
**(a)** The sequencing depth of CRNETs. **(b)** Genome-wide SNVs and INDELs discovered. From the outer circle to the inner circle: panel I: chromosome number and location; panel II: the point substitution variant (SNV): red C>T/G>A, blue C>A/G>T, black C>G/G>C, green T>C/A>G, gray T>A/A>T, pink T>G/A>C, and VAF height; panel III: INDEL: yellow for insertion, purple for deletion.

SUPPLEMENTARY FIGURE S5
**(a)** Histograms of the number of SNVs and INDELs in 24 CRNETs samples. **(b–d)** Histograms of the number of SNVs and INDELs, G1 **(b)**, G2 **(c)**, G3 **(d)**.

SUPPLEMENTARY FIGURE S6 Characteristics of potential somatic SNVs. **(a)** Proportions of six SNV types in potential somatic SNVs. **(b)** Comparison between the observed distribution of the somatic SNVs across the 96 possible mutation types and the summation of the distributions of the decomposed signatures. **(c–e)** Interdistance of the six types of SNVs in G1 **(c)**, G2 **(d)**, G3 **(e)**.

SUPPLEMENTARY FIGURE S7
**(a–c)** Boxplot of the fraction of the types of SNVs, including 6 types of SNVs, G1 **(a)**,G2 **(b)**, G3 **(c)**. **(d–f)** Boxplot of the fraction of the types of SNVs, including transitions vs transversions, G1 **(d)**, G2 **(e)**, G3 **(f)**.

SUPPLEMENTARY FIGURE S8
**(a)** Histograms of the number of detailed mutation classifications in 24 CRNETs samples. **(b–d)** Histograms of the number of detailed mutation classifications, G1 **(b)**, G2 **(c)**, G3 **(d)**.

SUPPLEMENTARY FIGURE S9
**(a)** Pathway enrichment analysis of genes mutated specifically in G1/G2. **(b)** Pathway enrichment analysis of genes mutated specifically in G2/G3.The length of the column represents the -log10(p-value) of the enriched pathways. The number on the right side of the column represents the number of mutated genes detected in the pathway. **(c)** Pathway enrichment analysis of genes mutated in both G1/G2/G3. **(d)** Pathway enrichment analysis of SMG in G1/G2. **(e)** Pathway enrichment analysis of SMG in G2/G3.

SUPPLEMENTARY FIGURE S10
**(a,b)** Overlap of genes about disease-related variants in G1/G2 **(a)**, G2/G3 **(b)**. **(c,d)**. Results of disease-related mutations pathway enrichment analysis on the genes in G1/G2 **(c)** and G2/G3 **(d)**.

SUPPLEMENTARY FIGURE S11
**(a)** Detailed information on the mutation locations of the frequently mutated gene in clinically relevant genetic mutations in G1,G2,and G3. **(b)** Detailed information on the mutation locations of the TP53 in clinically relevant genetic mutations in G3.

SUPPLEMENTARY TABLE S1 Summary of HYDIN mutation frequencies across from public databases.

SUPPLEMENTARY TABLE S2 Expression profile of FAT4 across normal tissues from the GTEx database.

SUPPLEMENTARY TABLE S3 Recurrent copy number alterations identified in the CRNET cohort.
