# Supplementary figures and images for: Comprehensive genomic profiling of neuroendocrine neoplasms of the colorectum
Source: Front Genet. 2026 May 12;17:1792341. doi: 10.3389/fgene.2026.1792341 (PMC13200827; doi:10.3389/fgene.2026.1792341)

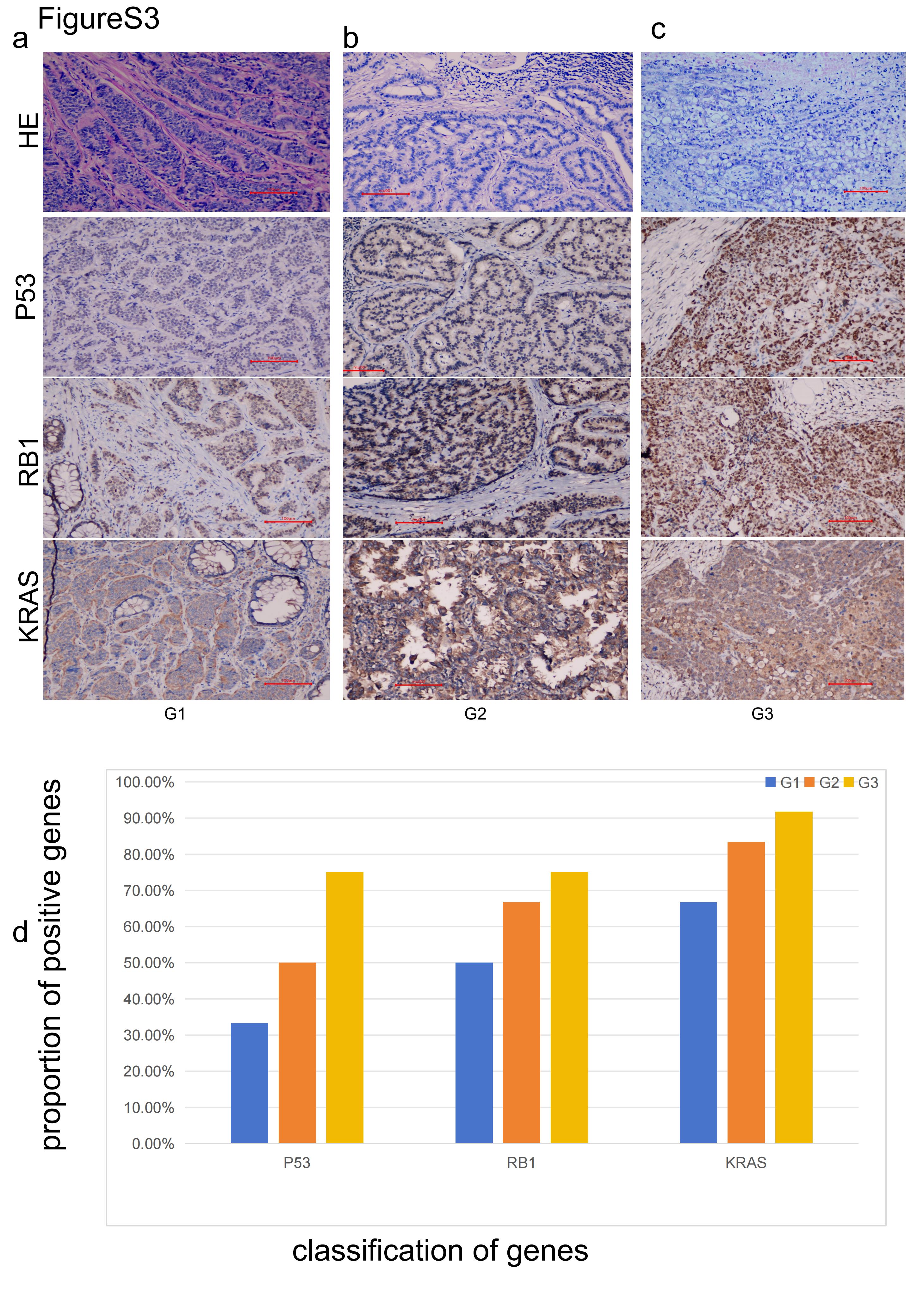

Supplement: Supplementary file 2 [file Image3.jpeg]

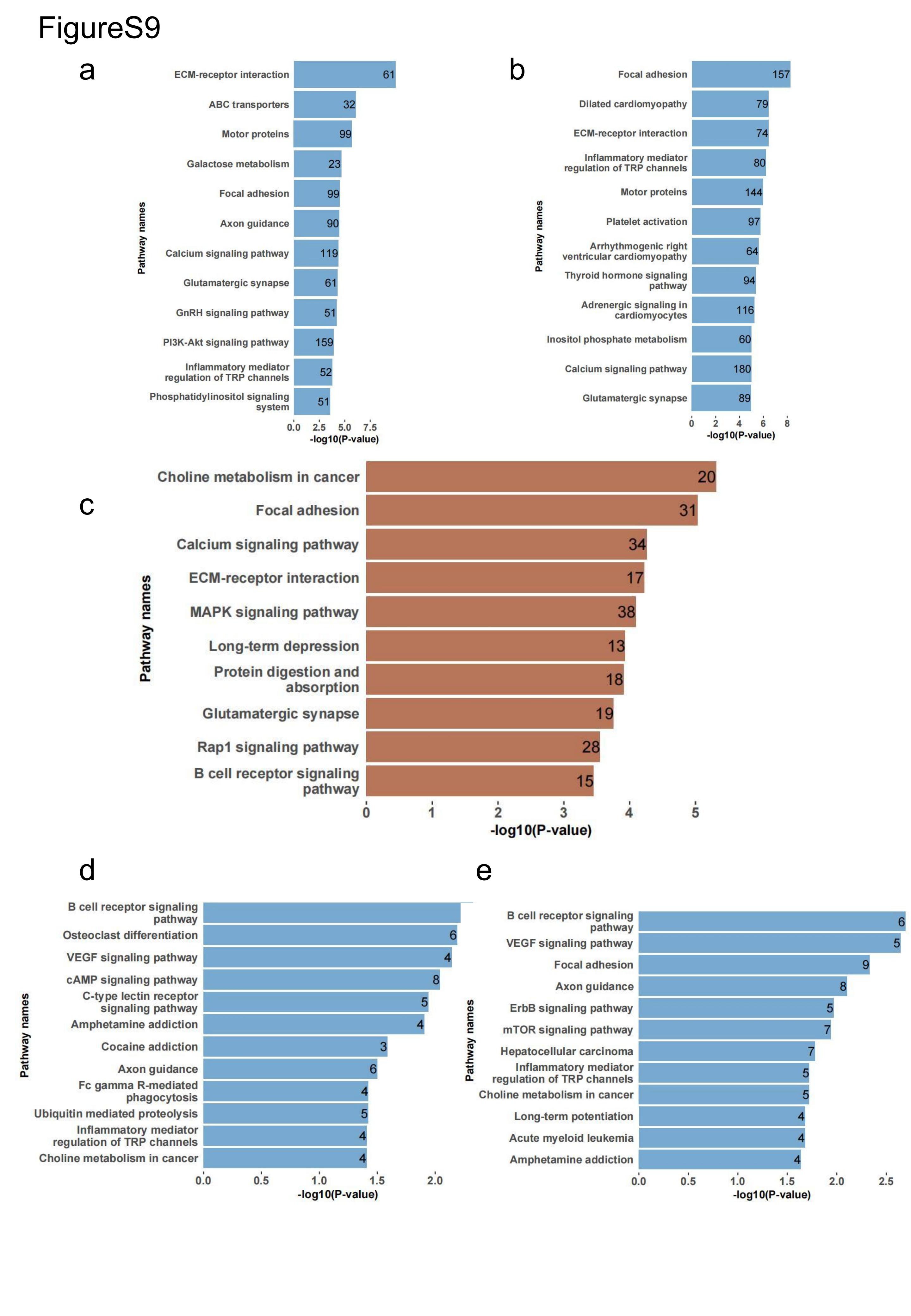

Supplement: Supplementary file 4 [file Image9.jpeg]

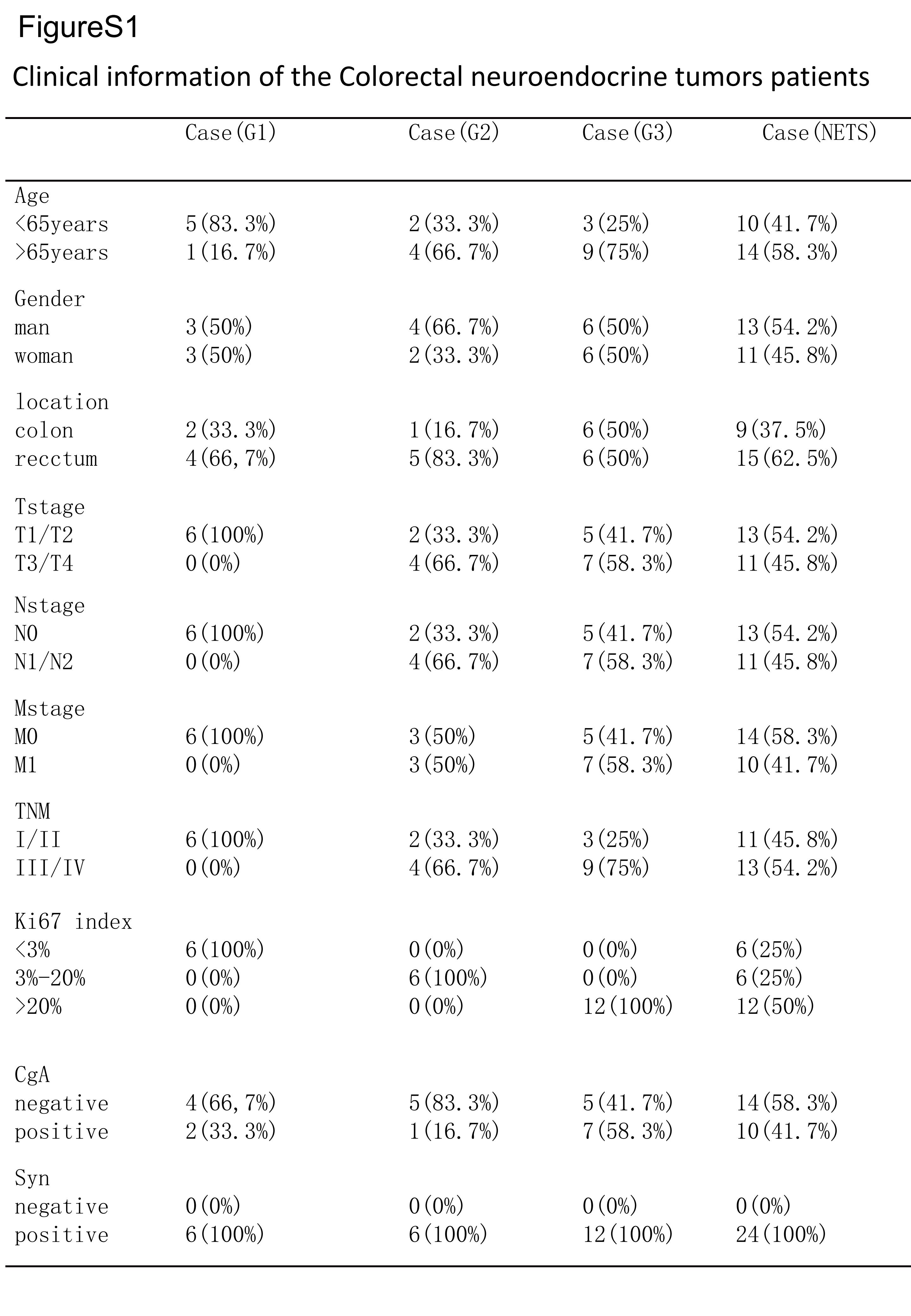

Supplement: Supplementary file 5 [file Image1.jpeg]

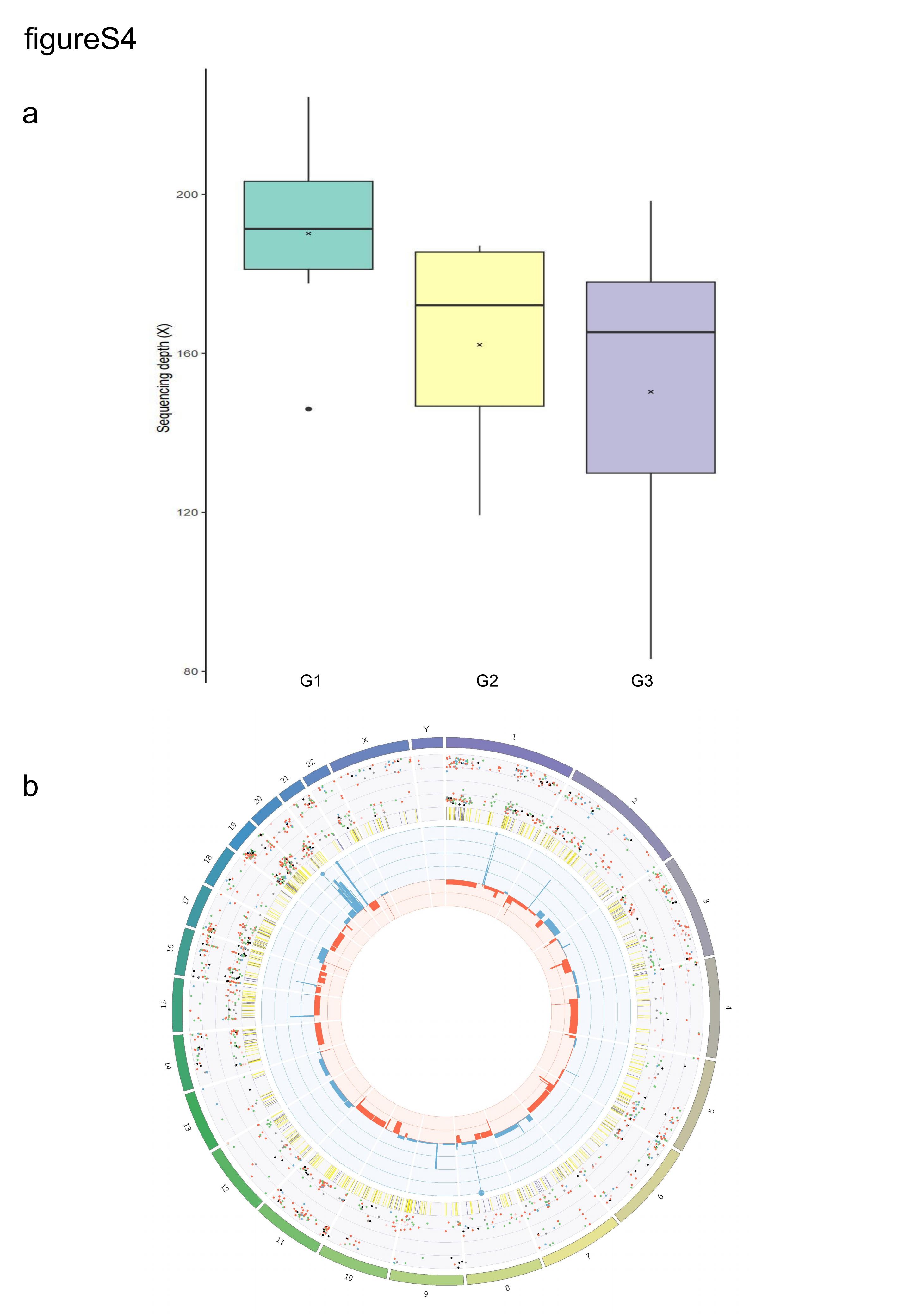

Supplement: Supplementary file 6 [file Image4.jpeg]

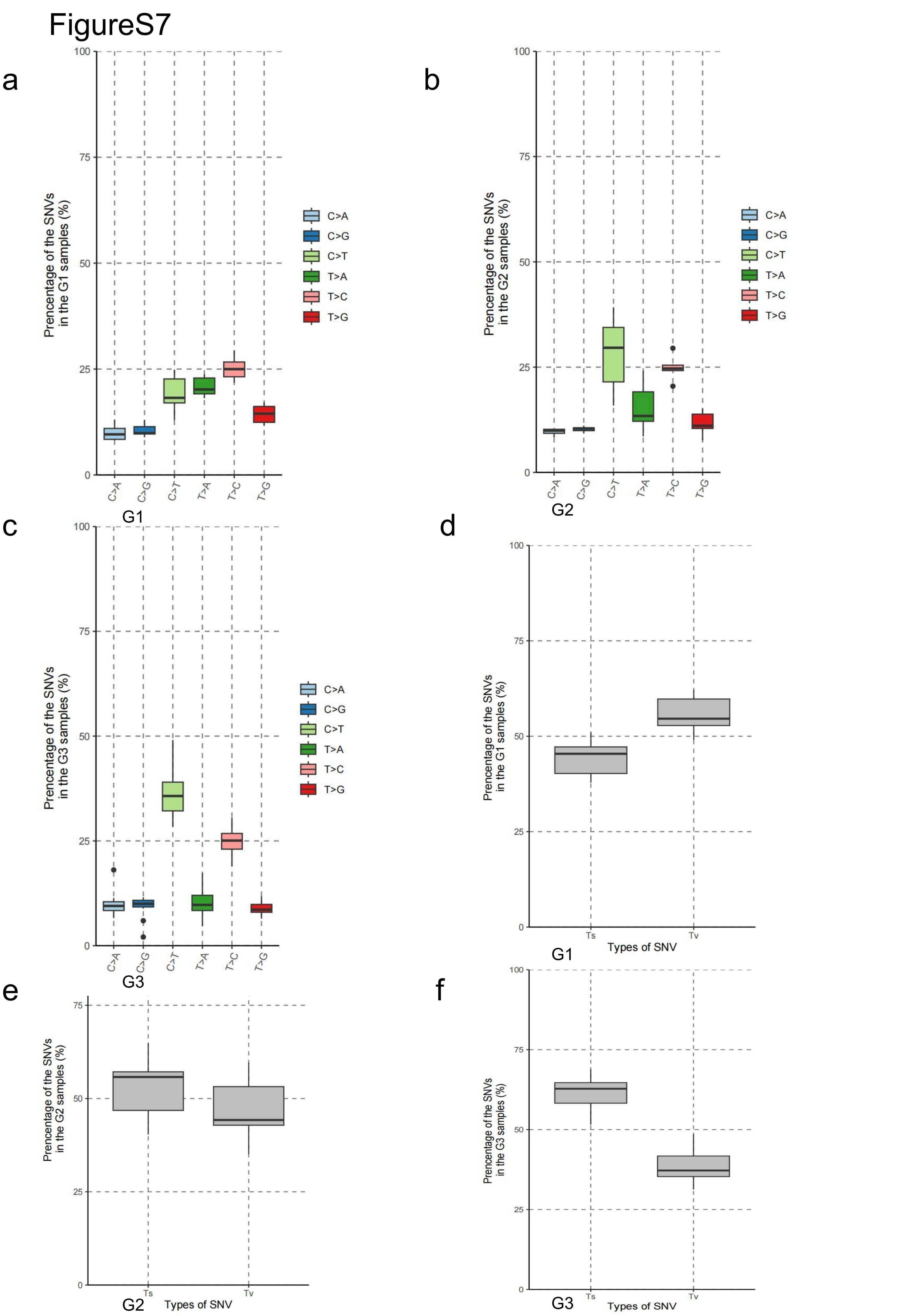

Supplement: Supplementary file 7 [file Image7.jpeg]

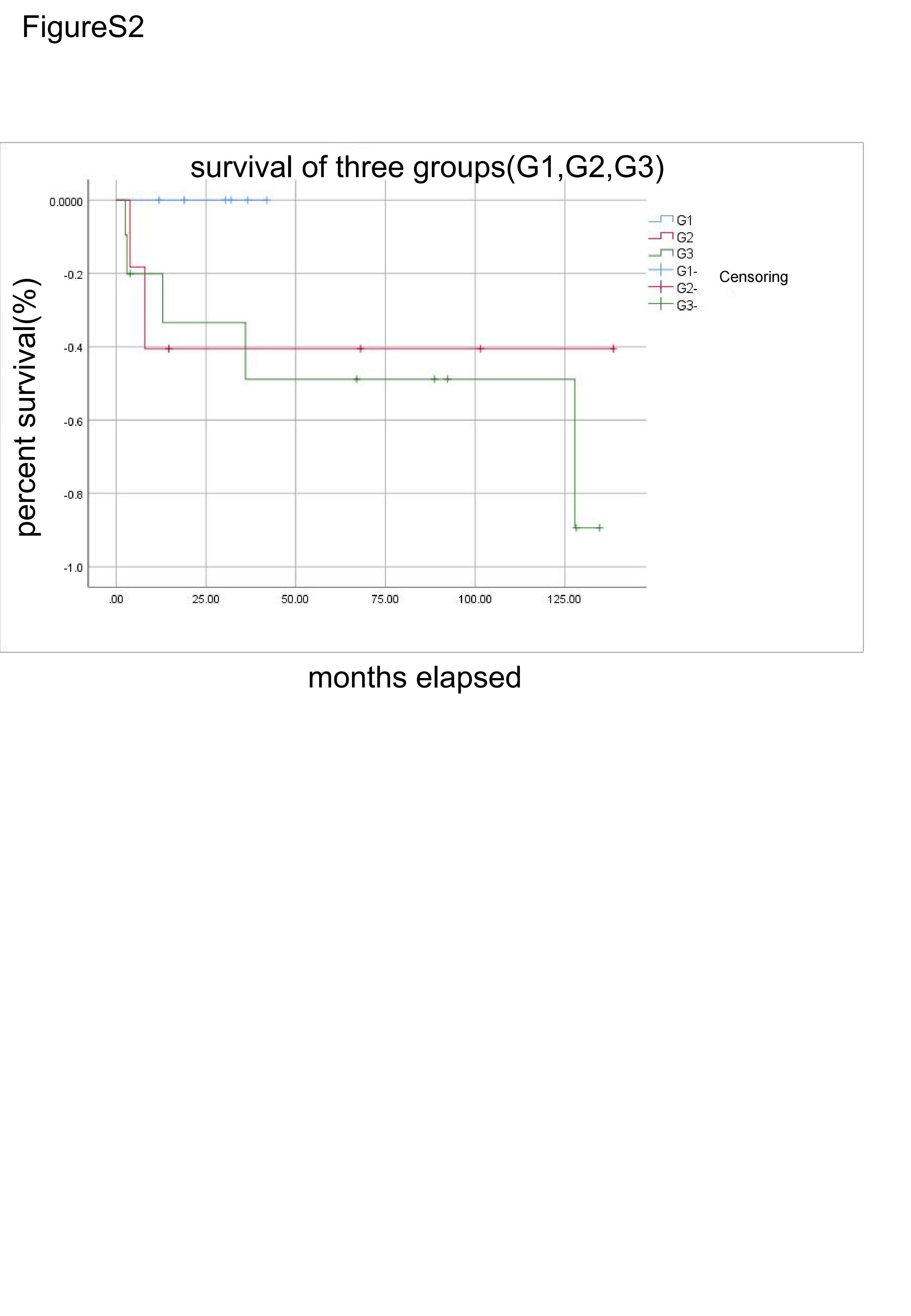

Supplement: Supplementary file 8 [file Image2.jpeg]

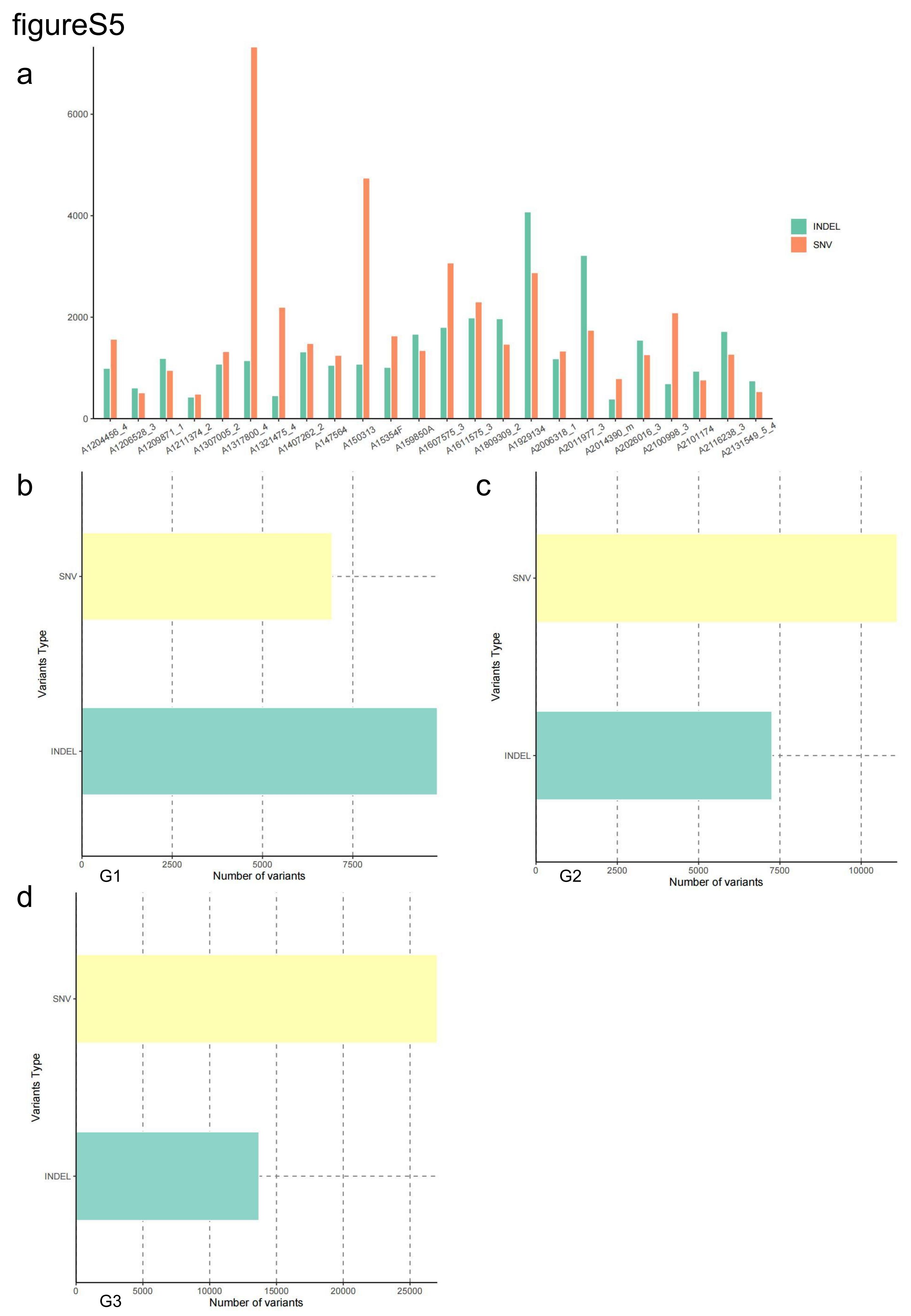

Supplement: Supplementary file 9 [file Image5.jpeg]

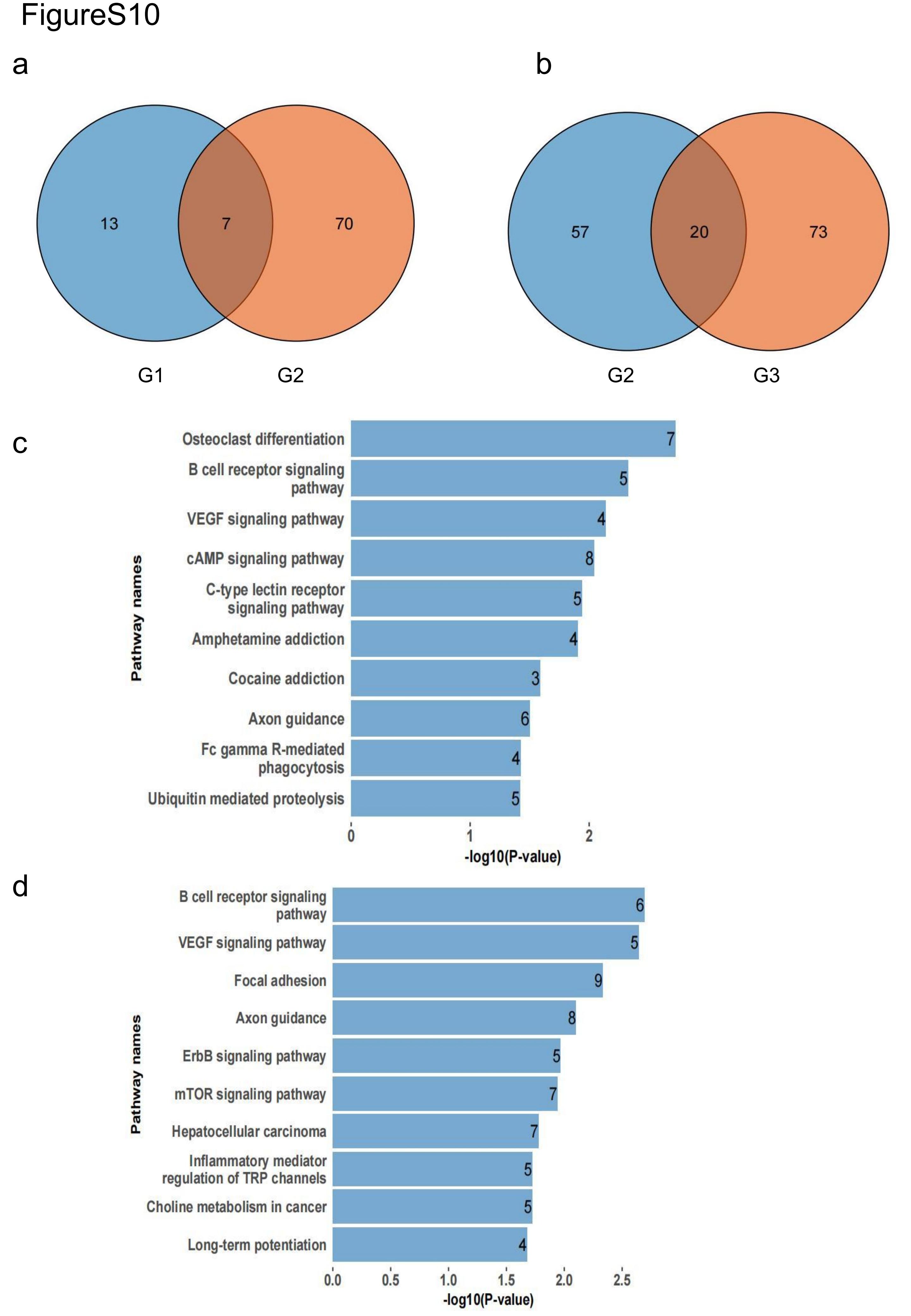

Supplement: Supplementary file 10 [file Image10.jpeg]

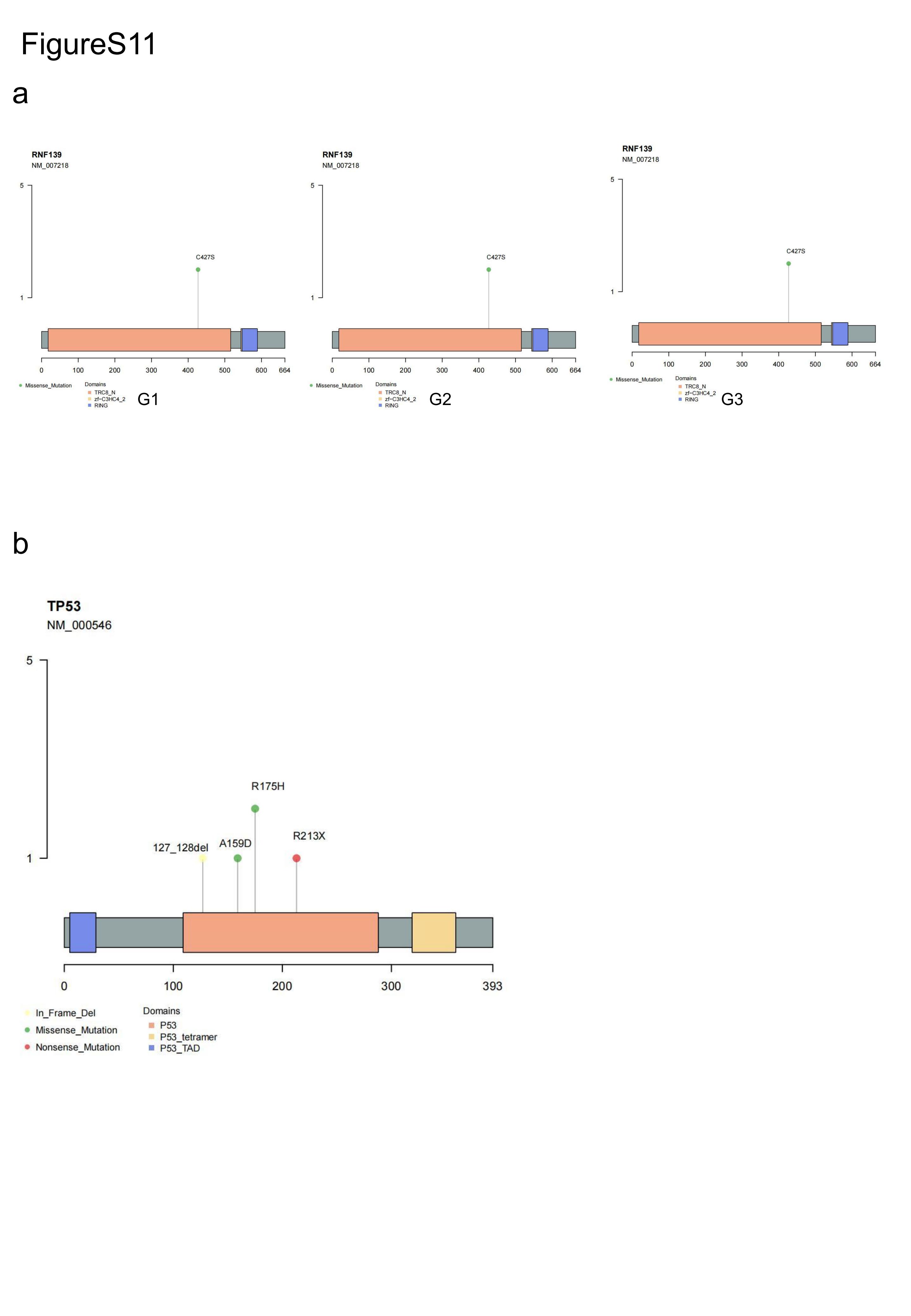

Supplement: Supplementary file 11 [file Image11.jpeg]

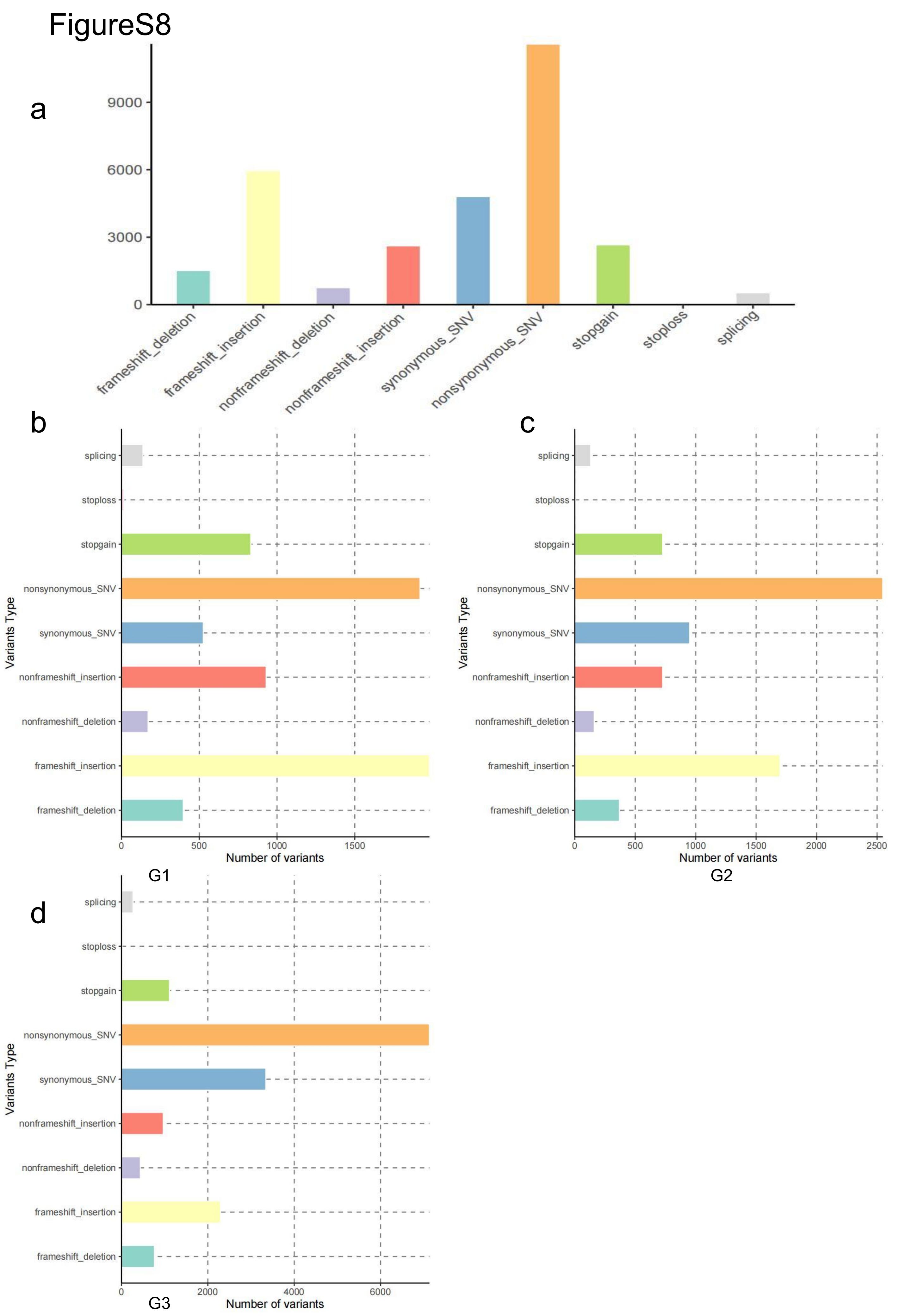

Supplement: Supplementary file 13 [file Image8.jpeg]

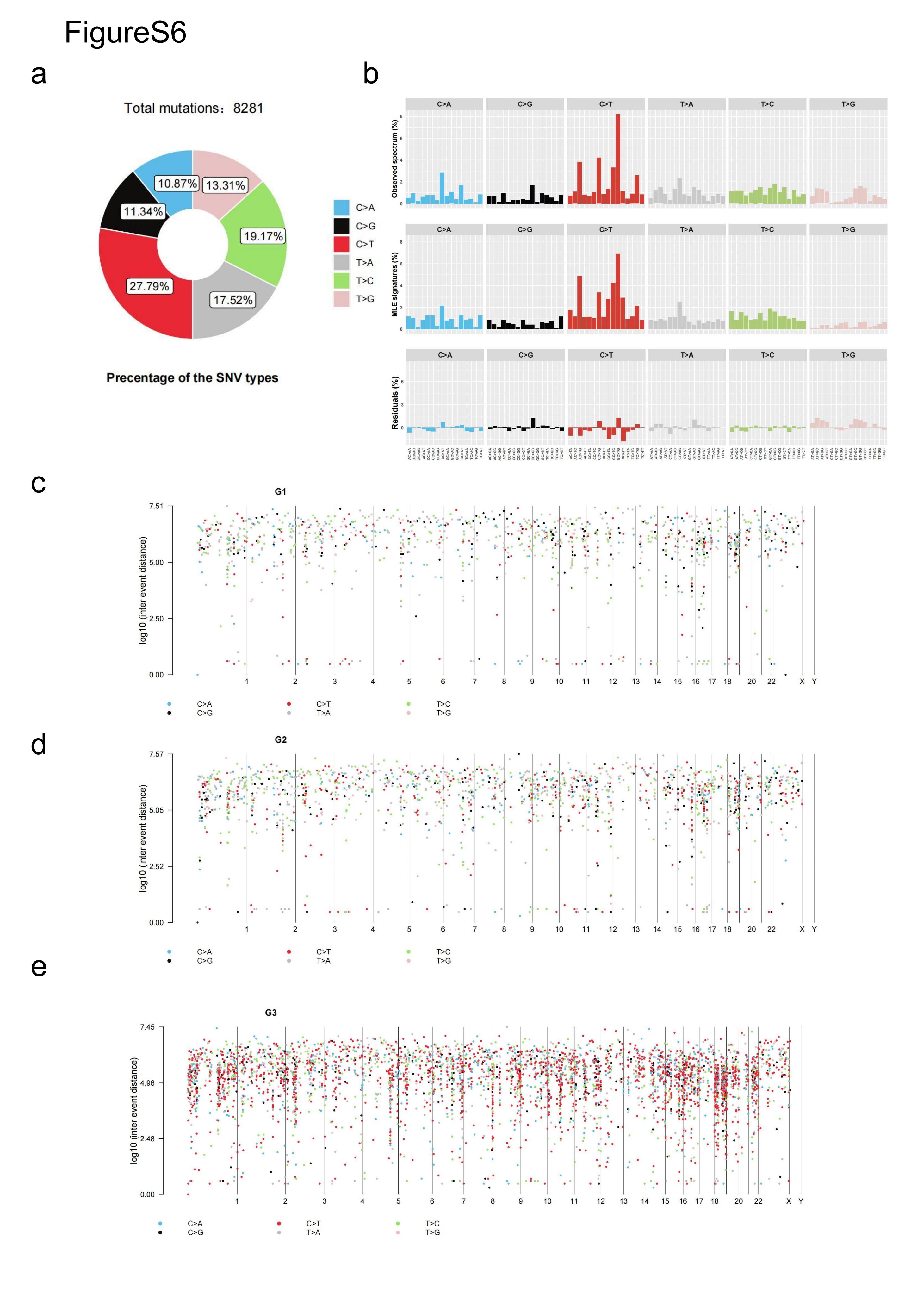

Supplement: Supplementary file 14 [file Image6.jpeg]
